# Development and validation of web-based, interpretable predictive models for sepsis and mortality in extensive burns

**DOI:** 10.3389/fcimb.2025.1586087

**Published:** 2025-08-18

**Authors:** Shi-Qi Wang, Kan Qiu, Qi-Rui Zheng, Bing-Jie Zhou, Ming-Yu Li, Hai-Yan Zhong, Yong Chen, Si-Ming Yuan

**Affiliations:** ^1^ Department of Burns and Plastic Surgery, Jinling Hospital, Jinling Clinical Medical College, Nanjing University of Chinese Medicine, Nanjing, Jiangsu, China; ^2^ Department of Burns and Plastic Surgery, Jinling Hospital, Affiliated Hospital of Medical School, Nanjing University, Nanjing, Jiangsu, China; ^3^ Department of Burns and Plastic Surgery, Anqing Petrochemical Hospital, Nanjing Drum Tower Hospital Group, Anqing, Anhui, China; ^4^ School of Computer Science, Peking University, Beijing, China; ^5^ Southeast University School of Medicine, Nanjing, Jiangsu, China

**Keywords:** extensive burns, machine learning, sepsis, mortality, predictive model, SHAP, XGBoost, GBT

## Abstract

**Background:**

Burn injuries are a common cause of trauma globally, with extensive burns (≥ 50% total body surface area burned) associated with high rates of sepsis and mortality. This study aims to identify risk factors associated with sepsis and mortality in extensively burned patients and to develop accurate, interpretable predictive models via machine learning algorithms.

**Methods:**

A retrospective cohort study was conducted utilizing data from two Burn Critical Care Units in Eastern China from 2012-2023. A total of 237 patients with extensive burns were included. We applied ten machine learning algorithms, including random forest, gradient boosting tree (GBT), and logistic regression, to predict sepsis and mortality. The models were evaluated via AUC, precision, recall, accuracy, and F1 score, and were compared with the SOFA score performance. Model interpretability was enhanced via SHapley Additive exPlanations (SHAP).

**Results:**

The key predictive factors for sepsis included the SOFA score, new onset shock, albumin, blood urea nitrogen (BUN), third-degree burned area, TBSA burned, white blood cell count, and inhalation injury. For mortality, the key predictive factors included alanine aminotransferase (ALT), the SOFA score, type of burn, new onset shock, third-degree burn area, TBSA burned, and sepsis. The RF model demonstrated superior performance in predicting sepsis (AUC = 0.977, accuracy = 0.945, recall = 0.964, precision = 0.930, and F1 score = 0.945). For mortality prediction, the GBT model yielded the highest AUC of 0.981 (accuracy = 0.952, recall = 0.965, precision = 0.942, and F1 score = 0.953). The sepsis prediction model outperformed the SOFA-based logistic regression model. Web-based calculators were developed to aid clinical decision-making.

**Conclusion:**

Machine learning models, RF and GBT, demonstrate strong predictive ability for sepsis and mortality in extensive burn patients. The application of SHAP enhances model transparency, facilitating clinical interpretation and early intervention. Two web-based calculators can guide intensive care strategies and improve patient outcomes.

## 1 Introduction

Burns are one of the most common and severe forms of trauma worldwide, with approximately 330,000 deaths annually attributed to burns (Organization, W.H, 2002). In developing countries, burns predominantly affect young individuals and the working population, imposing a significant burden on healthcare systems, labor markets, and economic development. Extensive burns (with a burn area ≥ 50% of the total body surface area, TBSA) cause profound disruption of the skin barrier, leading to vascular leakage, hypovolemic shock, immune dysregulation, and metabolic disturbances. These systemic responses significantly increase the susceptibility to sepsis (Cato et al., 2018; Torres et al., 2021; Yang et al., 2023). Compared with patients sustaining burns of 20–50% TBSA, those with burns exceeding 50% are at particularly high risk due to the critical shortage of autologous donor skin, which delays wound closure and prolongs exposure to pathogens (Zhang et al., 2021). Reports indicate that the mortality rate associated with burns complicated by sepsis can be as high as 20.3% (Yoon et al., 2018). Early identification of sepsis risk and prompt administration of appropriate antimicrobial treatment can effectively improve prognosis and even reduce mortality (Li et al., 2022). In addition to sepsis, large burns frequently precipitate systemic inflammatory response syndrome (SIRS), further promoting organ dysfunction, including cardiovascular collapse, acute respiratory distress syndrome (ARDS), and neurogenic shock (Raith et al., 2017). Identifying risk factors associated with mortality allows burn centers to quickly integrate resources for targeted, prioritized care.

Severe burns result in profound systemic responses, including sustained inflammation and metabolic disturbances, which complicate the early recognition of sepsis. Unlike earlier criteria that emphasize systemic inflammatory markers, such as those defined by the American Burn Association (ABA) (Greenhalgh et al., 2007) and the Mann-Salinas burn-specific predictors (Mann-Salinas et al., 2013; Yan et al., 2018), the Sepsis-3 consensus definition established in 2016 prioritizes organ dysfunction over inflammatory signs (Singer et al., 2016), representing a more appropriate approach for this patient population. In a comparative study, Yan et al. (2018) evaluated the diagnostic sensitivity of these three criteria in burn patients and found that Sepsis-3 demonstrated a sensitivity of 85%, which was higher than that of the ABA criteria (60%) and the Mann-Salinas model (20%), supporting its broader clinical applicability in this context.

In addition to sepsis, accurate prediction of mortality in patients with extensive burns remains a major clinical challenge. Several classical prognostic models have been developed to estimate mortality in burn patients, including the Baux score (age + %TBSA) (Baux, 1961), the Abbreviated Burn Severity Index (ABSI) (age, gender, inhalation injury, TBSA) (Tobiasen et al., 1982), and the Flame score (age, APACHE II, TBSA, gender) (Gomez et al., 2008). However, with advances in critical care, the relevance of these models has been increasingly questioned. For example, the ABSI associates female gender with worse outcomes, though recent evidence disputes this link (Ederer et al., 2019). The Baux score, being overly simplistic, lacks predictive accuracy (Liu et al., 2015), while the Flame score is too complex for routine bedside use without computational tools.

Machine learning is a subdiscipline of artificial intelligence that uses various mathematical algorithms to identify certain data patterns, thus making predictive conclusions about selected endpoints. Unlike traditional statistical models, machine learning can independently identify complex data relationships, overcome linear limitations, and ensure stability in high-dimensional datasets (Deo, 2015). Recent studies have utilized machine learning to develop predictive models for burn patients; for instance, Tran et al. developed an automated machine-learning platform to predict sepsis in burn patients using clinical data (Tran et al., 2020), while Jeon et al. developed a model leveraging immature granulocytes as biomarkers for sepsis prediction (Jeon et al., 2021). However, these studies mainly focus on general burn patients with smaller TBSA and use single algorithms without incorporating interpretability. There is no interpretable machine learning model specifically developed for clinical application in patients with extensive burn injuries.

In this study, our objective was to develop predictive models for sepsis and mortality in patients with extensive burns, based on data obtained within the first 24 hours of admission. We conducted a retrospective study of patients with extensive burns admitted to two Burn Critical Care Units in Eastern China. Using ten commonly applied machine learning algorithms, we developed predictive models to identify the best-performing approaches. Additionally, we compared the sepsis prediction model we developed with the SOFA score prediction using receiver operating characteristic (ROC) curve analysis to further validate its discrimination. To interpret the results of the machine learning models, we combined advanced machine learning algorithms with a method based on SHapley Additive exPlanations (SHAPs). SHAP is a popular machine learning technique that can provide a deeper understanding of the complex relationships between features and predictions (Lundberg et al., 2018). Intuitive explanations help clinicians comprehensively understand how the developed models make specific predictions, thereby increasing opportunities for early intervention. Finally, we created web-based calculators for the optimal models to facilitate their use by clinical practitioners.

## 2 Methods

### 2.1 Study population

A 12-year retrospective, dual-center dataset was established, covering patients with extensive burns who were admitted to two Burn Critical Care Units, Jinling Hospital Burn Critical Care Unit and Anqing Petrochemical Hospital Burn Critical Care Unit, in Eastern China from January 2012 to December 2023. The inclusion and exclusion criteria were as follows:

**Table:** 

Inclusion criteria	Exclusion criteria
1. Total body surface area (TBSA) burned ≥ 50%	1. Incomplete data exceeding 30% or refusal to participate
2. Age > 18 years	2. Pre-existing critical illnesses, such as malignant tumors, severe autoimmune diseases, or organ failure
	3. Hospitalization duration ≤ 24 hours

**Figure 1:**
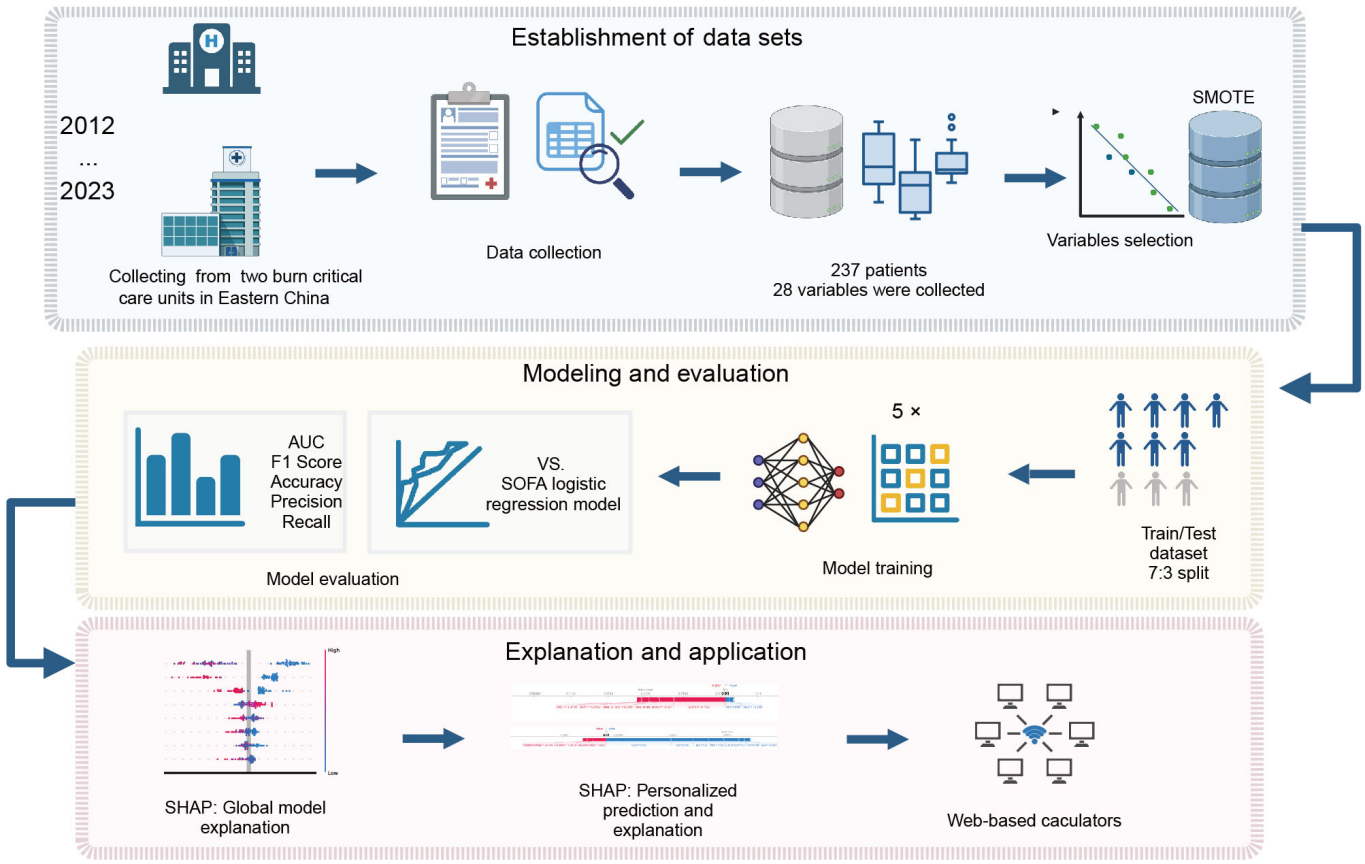
Flowchart of the study. SMOTE, synthetic minority over-sampling technique; SOFA, sequential organ failure assessment; AUC, area under the curve; SHAP, SHapley additive exPlanations.

This study received approval from the Institutional Review Board of Jinling Hospital (Approval No. 2023-LLSC-026). Given the retrospective study design and anonymized clinical data, informed consent from participants was waived. The flowchart of the study was presented in **Figure 1**. The process of data selection is illustrated in **Supplementary Figure S1** (see the appendix in the electronic **Supplementary Material**).

### 2.2 Outcomes and predictors

#### 2.2.1 Outcomes

Sepsis: Sepsis refers to an occurrence during hospitalization. The presence of sepsis and septic shock was determined according to the Sepsis 3.0 criteria, which require evidence of infection combined with an increase in the SOFA score of ≥ 2 points (Singer et al., 2016). For consistency across the study period (2013–2022), all sepsis cases were retrospectively identified using the Sepsis-3 definition by trained clinicians during data collection in 2022–2024.

Mortality: Mortality refers to in-hospital death occurring beyond 24 hours after admission. Patients were classified as either survivors or non-survivors accordingly.

#### 2.2.2 Predictors

The following variables collected upon admission were considered as potential predictors, categorized and discussed as follows:

1. Demographic Information: age, sex.
2. Clinical Characteristics at Admission: injury to hospital time, type of burn, TBSA burned, third-degree burned area (%TBSA), open decompression, tracheotomy, presence of inhalation injury, shock status, history of hypertension or diabetes mellitus, Sequential Organ Failure Assessment (SOFA) score: emphasized as a key prognostic indicator, providing a quantitative measure of organ dysfunction severity.
3. Treatment Factors: fluid resuscitation, primary treatment.
4. Laboratory Data: White Blood Cell (WBC) count, Red Blood Cell (RBC) count, hemoglobin; platelet count, Aspartate Aminotransferase (AST), Alanine Aminotransferase (AST), Indirect Bilirubin (IBIL), Direct Bilirubin (DBIL), Total Bilirubin (TBIL), Albumin (ALB), Globulin (GLB), Blood Urea Nitrogen (BUN), Serum Creatinine (SCR), Potassium (K^+^), Sodium (Na^+^), Chloride (Cl^-^), Calcium (Ca^2+^), Procalcitonin (PCT).

Both categorical and continuous variables were included to maximize the available information and capture diverse aspects of the patient’s condition.

### 2.3 Data processing and handling

The data were collected using Microsoft Excel (Redmond, Washington, USA) and processed with Python 3.9. Categorical variables were presented as counts (percentages) and analyzed using the chi-square test or Fisher’s exact test when appropriate. Otherwise, variables were expressed as means ± standard deviations and analyzed using the independent-samples t-test for normally distributed data; otherwise, the Mann-Whitney U test was applied. The overall rate of missing data was less than 5%. For variables with missing values, multiple imputations were used for numerical variables and mode imputation for categorical variables, assuming data were missing at random.

The dataset included 162 patients (68.4%) who developed sepsis and 75 patients (31.6%) who did not. Additionally, 199 patients survived (83.9%), whereas 38 (16.1%) died. The significant imbalance in the outcome categories posed a challenge for model prediction accuracy. To address this, we used the synthetic minority oversampling technique (SMOTE), which generates synthetic samples by interpolating between nearest neighbors within the minority class. This approach helps balance the data distribution and improves the model’s prediction capabilities for the minority class (Chawla et al., 2002; Haibo and Garcia, 2009). Variables with a *P* value < 0.001 in univariate regression analysis were selected as candidate factors for constructing predictive models.

### 2.4 Development of ten machine learning models

The entire dataset was randomly divided into a training set and a validation set at a 7:3 ratio, where the training set was used to construct the predictive models, and the validation set was used to evaluate model performance.

Ten commonly used machine learning algorithms were employed to establish prediction models, including Random Forest (RF), Multilayer Perceptron (MLP), Adaptive Boosting (AdaBoost), Logistic Regression (LR), Extreme Gradient Boosting (XGBoost), Naive Bayes (NB), Gradient Boosting Tree (GBT), Support Vector Machine (SVM), Decision Tree (DT), and K-Nearest Neighbor (KNN) methods. Among these, RF, AdaBoost, GBT, and XGBoost are ensemble classifiers, while the rest are single classifiers. During the training process, 5-fold cross-validation combined with grid search was applied to optimize the hyperparameters for each model, ensuring robustness and generalizability.

### 2.5 Model evaluation

The models’ performance was evaluated using several metrics, including accuracy, precision, recall, F1 score, and AUC (area under the curve). Higher scores closer to 1 indicate better predictive performance. ROC curves were used to graphically illustrate the discriminative power of the model. Furthermore, the ROC curve of the best-performing machine learning model was compared with that of a logistic regression model based solely on the SOFA score.

### 2.6 Model interpretability

To improve the interpretability of the optimal model, SHapley Additive exPlanations (SHAPs) were employed. SHAP values provide localized explanations, quantifying each variable’s impact on individual predictions (Lundberg et al., 2018). Originating from cooperative game theory, SHAP values represent the average marginal contribution of a variable across all possible combinations. For each individual prediction, the SHAP value indicates whether a variable contributes positively or negatively to the predicted outcome.

### 2.7 Web-based calculators

Two user-friendly web-based calculators were developed using Streamlit (https://github.com/streamlit/streamlit). Streamlit is an open-source library that enables researchers to create web applications with only a few lines of Python code, without requiring expertise in front-end development.

## 3 Results

### 3.1 Data characteristics

After applying the inclusion and exclusion criteria, data from 237 patients with extensive burns were included in the final model development (**Figure 1**). The dataset comprised 147 male and 90 female patients. The baseline characteristics, clinical features, treatment interventions, and initial laboratory results upon admission were summarized in **Table 1**. Additional in-hospital information, including wound culture, blood culture, catheter culture, length of stay, and multidrug-resistant (MDR), is provided in **Supplementary Table S1**.

**Table 1:** Differences in demographic and clinical characteristics between sepsis and non-sepsis groups, as well as between the survived and deceased groups.

Variables	n (%)					
	Non-sepsis	Sepsis	*p-*values	Survived	Deceased	*p-*values
Sex			0.385			1
Female	58 (35.80)	32 (42.67)		76 (38.19)	14 (36.84)	
Male	104 (64.20)	43 (57.33)		123 (61.81)	24 (63.16)	
Age (y) (median, [IQR])	52.00 [36.25, 62.75]	49.00 [40.00, 56.00]	0.399	51.00 [35.00, 61.00]	52.50 [44.25, 58.75]	0.297
Injury to hospital time (days)	2.95 ± 0.98	2.8 ± 1.18	0.36	2.87 ± 1.06	3.09 ± 0.94	0.192
TBSA burned (%)	56.07 ± 5.85	61.36 ± 9.71	<0.001	57.23 ± 7.37	60.45 ± 8.79	0.04
Third-degree burned area (%)	9.81 ± 12.93	18.76 ± 19.75	<0.001	10.54 ± 11.11	23.63 ± 28.47	0.008
Fluid resuscitation			0.424			0.318
No	118 (72.84)	59 (78.67)		146 (73.37%)	31 (81.58%)	
Yes	44 (27.16)	16 (21.33)		53 (26.63%)	7 (18.42%)	
Primary treatment			0.28			0.011
No treatment	102 (62.96)	52 (69.33)		130 (65.33%)	24 (63.16%)	
Local treatment	22 (13.58)	10 (13.33)		23 (11.56%)	9 (23.68%)	
Fluid resuscitation	38 (23.46)	12 (16.00)		46 (23.12%)	4 (10.53%)	
Comprehensive treatment	0 (0.00)	1 (1.33)		0 (0.0%)	1 (2.63%)	
Type of burn			0.574			0.006
Flame burn	121 (74.69)	58 (77.33)		157 (78.89)	22 (57.89)	
Scald	21 (12.96)	8 (10.67)		20 (10.05)	9 (23.68)	
Chemical burn	13 (8.02)	3 (4.00)		14 (7.04)	2 (5.26)	
Explosion	3 (1.85)	2 (2.67)		2 (1.01)	3 (7.89)	
Electrical injury	4 (2.47)	4 (5.33)		6 (3.02)	2 (5.26)	
Open decompression			0.227			0.16
No	158 (97.53)	70 (93.33)		193 (96.98)	35 (92.11)	
Yes	4 (2.47)	5 (6.67)		6 (3.02)	3 (7.89)	
Tracheotomy			0.142			0.299
No	130 (80.25)	53 (70.67)		151 (75.88)	32 (84.21)	
Yes	32 (19.75)	22 (29.33)		48 (24.12)	6 (15.79)	
Inhalation injury			0.007			0.355
No	115 (70.99)	39 (52.00)		132 (66.33)	22 (57.89)	
Yes	47 (29.01)	36 (48.00)		67 (33.67)	16 (42.11)	
SOFA (median, [IQR])	0.00 [0.00, 1.00]	2.00 [2.00, 3.00]	<0.001	1.00 [0.00, 2.00]	2.00 [1.00, 2.00]	0.003
Diabetes			0.22			0.25
No	155 (95.68)	68 (90.67)		189 (94.97)	34 (89.47)	
Yes	7 (4.32)	7 (9.33)		10 (5.03)	4 (10.53)	
Hypertension			0.227			1
No	158 (97.53)	70 (93.33)		191 (95.98)	37 (97.37)	
Yes	4 (2.47)	5 (6.67)		8 (4.02)	1 (2.63)	
New onset shock			<0.001			<0.001
No	161 (99.38)	38 (50.67)		179 (89.95)	20 (52.63)	
Yes	1 (0.62)	37 (49.33)		20 (10.05)	18 (47.37)	
Sepsis						<0.001
No	162 (100)	0 (0)		148 (74.37)	14 (36.84)	
Yes	0 (0)	75 (100)		51 (25.63)	24 (63.16)	
LOS of ICU (days)	41.70 ± 24.92	52.60 ± 30.03	0.006	48.55 ± 27.21	27.71 ± 18.46	<0.001
WBC (×10^9^/L)	20.09 ± 8.55	25.18 ± 11.53	<0.001	21.5 ± 9.62	22.74 ± 11.09	0.521
RBC (×10^12^/L)	5.0 ± 0.83	5.27 ± 0.83	0.019	5.09 ± 0.87	5.07 ± 0.66	0.87
HB (g/L)	149.32 ± 23.97	153.8 ± 22.4	0.163	151.12 ± 23.99	148.71 ± 21.14	0.531
PLT (×10^9^/L)	231.92 ± 84.28	244.56 ± 112.38	0.387	235.65 ± 95.76	237.32 ± 85.54	0.915
ALT (U/L)	33.44 ± 66.2	58.07 ± 108.93	0.073	28.58 ± 54.33	107.47 ± 149.93	0.003
AST (U/L)	53.1 ± 94.77	76.56 ± 139.16	0.188	52.59 ± 89.7	102.11 ± 182.75	0.11
IBIL (μmol/L)	13.41 ± 7.6	20.44 ± 17.67	0.001	15.5 ± 11.65	16.34 ± 14.75	0.741
DBIL (μmol/L)	4.7 ± 3.19	5.71 ± 4.38	0.077	5.25 ± 3.6	3.81 ± 3.57	0.026
TBIL (μmol/L)	52.63 ± 8.7	49.23 ± 9.32	0.009	51.56 ± 9.13	51.53 ± 8.54	0.986
ALB (g/L)	33.28 ± 6.35	29.93 ± 6.99	<0.001	32.31 ± 6.74	31.72 ± 6.73	0.622
GLB (g/L)	19.34 ± 4.67	18.51 ± 4.39	0.19	19.02 ± 4.42	19.37 ± 5.46	0.709
BUN (mmol/L)	5.08 ± 1.39	6.41 ± 2.04	<0.001	5.45 ± 1.74	5.73 ± 1.73	0.376
SCR (μmol/L)	71.42 ± 28.68	107.13 ± 57.92	<0.001	81.92 ± 44.34	86.94 ± 38.92	0.479
Glucose (mmol/L)	12.35 ± 9.16	11.82 ± 7.01	0.624	12.25 ± 8.6	11.79 ± 8.22	0.753
K^+^ (mmol/L)	3.82 ± 0.52	3.8 ± 0.63	0.796	3.79 ± 0.56	3.93 ± 0.53	0.141
Na^+^ (mmol/L)	137.19 ± 3.32	138.47 ± 4.15	0.021	137.6 ± 3.77	137.57 ± 2.95	0.951
CI^-^ (mmol/L)	103.84 ± 3.96	104.97 ± 6.06	0.142	104.28 ± 4.68	103.76 ± 5.12	0.564
Ca^2+^ (mmol/L)	1.98 ± 0.21	1.95 ± 0.24	0.412	1.97 ± 0.23	1.97 ± 0.17	0.938
PCT (ng/mL)	3.01 ± 7.85	6.83 ± 15.38	0.045	4.35 ± 11.78	3.55 ± 4.32	0.465

SOFA, sequential organ failure assessment; WBC, white blood cells; RBC, red blood cells; HB, hemoglobin; PLT, platelet count; ALT, alanine aminotransferase; AST, aspartate aminotransferase; IBIL, indirect bilirubin; DBIL, direct bilirubin; TBIL, total bilirubin; ALB, albumin; GLB, globulin; BUN, blood urea nitrogen; SCR, serum creatinine; PCT, procalcitonin.

In comparison to the non-sepsis cohort, the sepsis group exhibited a significantly higher rate of inhalation injuries (48.00% *vs*. 29.01%, *P* = 0.007). The TBSA burned was greater in the sepsis group (61.36% *vs*. 56.07%, *P* < 0.001), with a notably greater proportion of III-degree burns (18.76% *vs*. 9.81%, *P* < 0.001). In terms of laboratory findings, the sepsis group presented higher elevated white blood cell counts (25.18 ± 11.53 *vs*. 20.09 ± 8.55, *P* < 0.001). Additionally, new onset shock was observed in 49.33% of patients with sepsis (n = 37), compared to only one case (0.62%) in the non-sepsis group (*P* < 0.001). Abnormal liver and kidney function, reflected in elevated TBIL, BUN, and SCR, was also more prevalent in the sepsis group. Lastly, the median SOFA score was significantly higher among sepsis patients [2.00 (IQR: 2.00–3.00) *vs*. 0 (IQR: 0.00–1.00)], suggesting its value in early prognostic assessment.

Comparisons between the mortality and survival groups revealed that extensive burns resulting from scalds (23.68% *vs*. 11.56%), explosions (7.89% *vs*. 3.02%) and electrical injuries (5.36% *vs*. 3.02%) were more prevalent in the mortality group. Additionally, the TBSA burned (60.45 ± 8.79% *vs*. 57.23 ± 7.37%, *P* = 0.04) and third-degree burned area (23.63 ± 28.47% *vs*. 10.54 ± 11.11%, *P* = 0.008) were notably greater. The mortality group presented a significantly greater incidence of new onset shock (47.37% *vs*. 10.05%, *P* < 0.001) and sepsis (63.16% *vs*. 25.63%, *P* < 0.001). Among the laboratory indicators, abnormal elevations in the liver enzyme ALT (107.47 ± 149.93 *vs*. 28.58 ± 54.33, *P* = 0.003) were more pronounced in the mortality group. The SOFA score also demonstrated a marked difference, with a median score of 2.00 (IQR: 1.00, 2.00) in the mortality group versus 1.00 (IQR: 0.00, 2.00) in the survival group, emphasizing its value as a predictive tool.

### 3.2 Features selected in the models

The data balanced via the SMOTE algorithm is shown in **Supplementary Table S2**. Using sepsis occurrence as the outcome variable, univariate regression analysis was conducted to identify significant features. The regression coefficients and odds ratios (ORs) with 95% confidence intervals for these predictors are summarized in **Table 2**. After highly correlated features were removed, 8 key variables (*P* < 0.005) were selected: TBSA burned, third-degree burned area, new-onset shock, WBC, ALB, the SOFA score, and inhalation injury.

**Table 2:** Features selected in models.

Sepsis				Mortality			
Variables	β	Odds ratio (95% CI)	*p*-values	Variables	β	Odds ratio (95% CI)	*p*-values
TBSA burned	0.091	1.096 (1.053, 1.14)	<0.001	Sepsis	1.604	4.975 (2.393, 10.343)	<0.001
Third-degree burned area	0.036	1.036 (1.016, 1.057)	<0.001	TBSA burned	0.047	1.048 (1.007, 1.092)	0.023
New onset shock	5.055	156.763 (20.847, 1178.803)	<0.001	Third-degree burned area	0.039	1.040 (1.020, 1.060)	<0.001
WBC	0.052	1.053 (1.023, 1.084)	<0.001	Type of burn (*vs*. Flame burn)			
ALB	-0.075	0.928 (0.89, 0.968)	0.001	Scald	1.167	3.211 (1.300, 7.934)	0.011
BUN	0.471	1.602 (1.33, 1.928)	<0.001	Chemical burn	0.019	1.019 (0.217, 4.791)	0.981
SOFA	2.024	7.57 (4.434, 12.923)	<0.001	Explosion	2.371	10.705 (1.693, 67.673)	0.012
Inhalation injury	0.815	2.26 (1.754, 2.223)	<0.005	Electrical injury	0.867	2.379 (0.452, 12.528)	0.307
				New onset shock	0.85	0.701 (0.655, 0.742)	<0.001
				ALT	1.587	0.830 (0.722, 0.902)	<0.001
				SOFA	0.843	0.699 (0.636, 0.755)	<0.001

TBSA, total body surface area; WBC, white blood cells; ALB, albumin; BUN, blood urea nitrogen; SOFA, sequential organ failure assessment. ALT, alanine aminotransferase.

For mortality as the outcome variable, univariate regression analysis was similarly conducted. After highly correlated variables were excluded, 7 significant predictors (*P* < 0.001) were identified: sepsis, TBSA burned, third-degree burned area, type of burn, new onset shock, ALT, and the SOFA score.

### 3.3 Model evaluation

**Figure 2:**
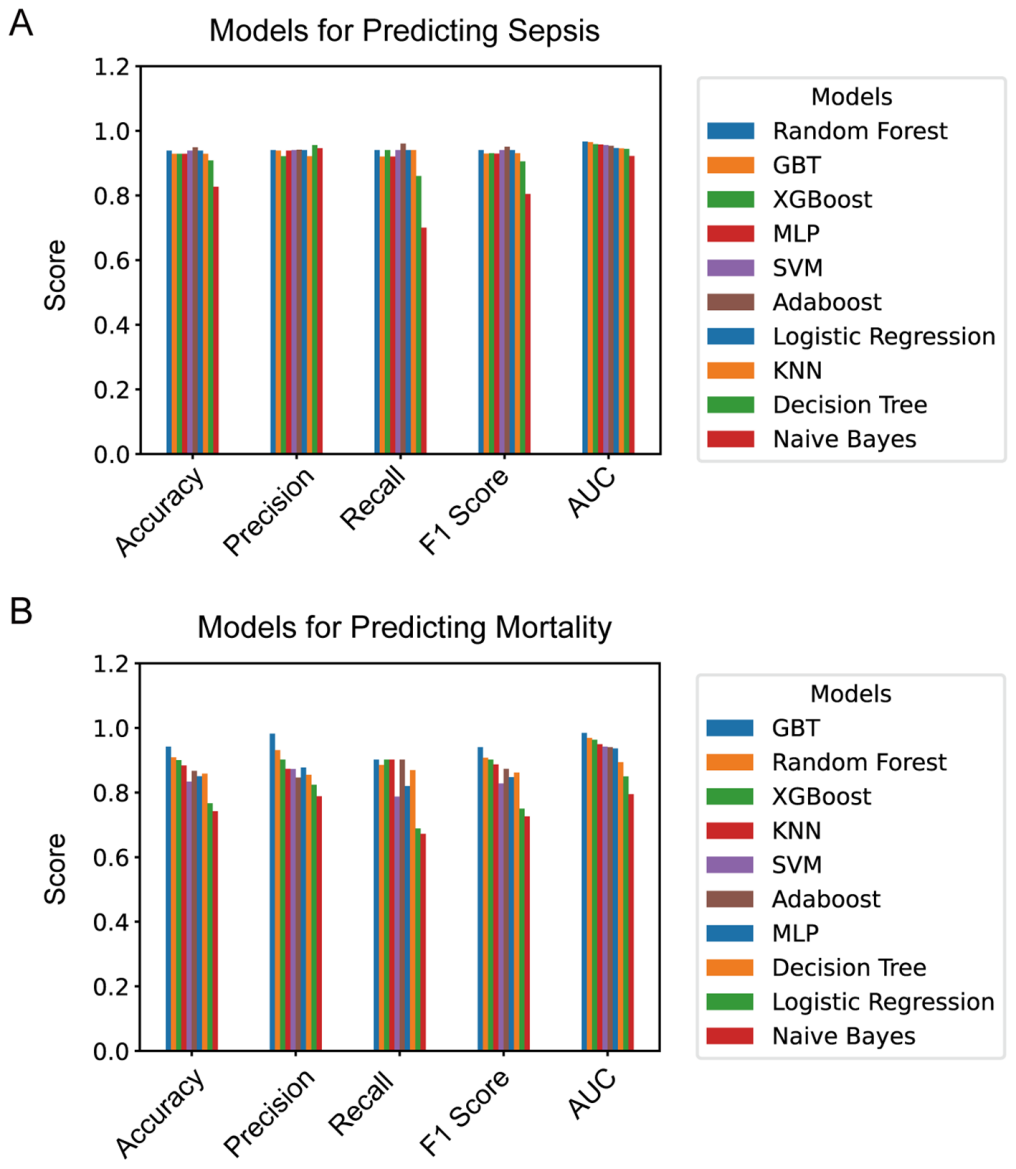
**(A)** Evaluation metrics of models for predicting sepsis; **(B)** evaluation metrics of models for predicting mortality. XGBoost, extreme gradient boosting; KNN, k-nearest neighbors; SVM, support vector machine; GBT, gradient boosting tree; Adaboost, adaptive boosting; MLP, multi-layer perceptron; AUC, area under the curve.

**Figure 3:**
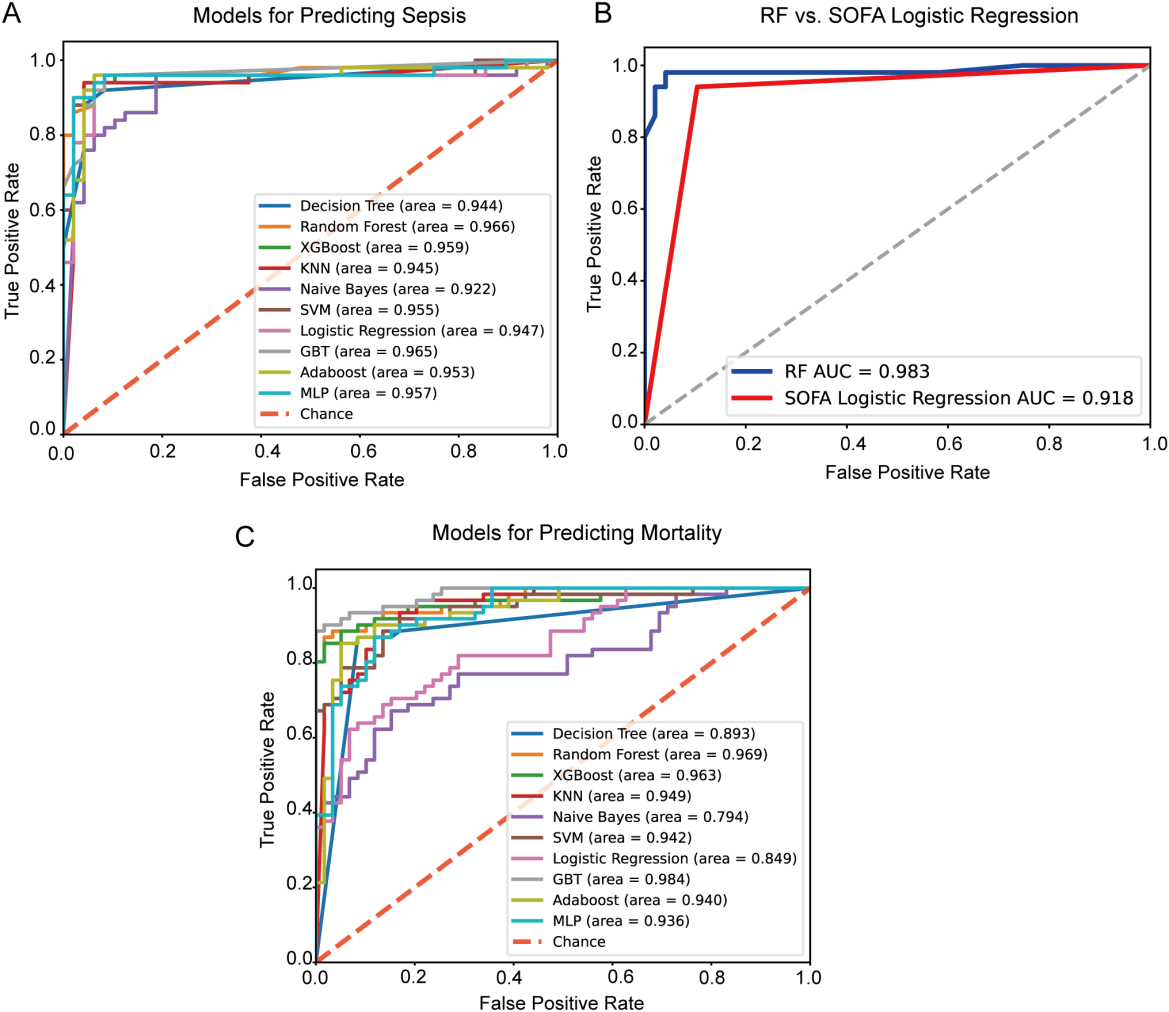
ROC curves. **(A)** ROC curves for predicting sepsis in extensive burn patients with machine learning algorithms; **(B)** The ROC curve comparison between the RF model and the SOFA logistic regression model for predicting sepsis in extensive burn patients; **(C)** ROC curves for predicting mortality in extensive burn patients with machine learning algorithms; **(B)** The ROC curve comparison between the XGBoost model and the SOFA logistic regression model for predicting mortality in extensive burn patients. ROC, receiver operating characteristic; XGBoost, extreme gradient boosting; KNN, k-nearest neighbors; SVM, support vector machine; GBT, gradient boosting tree; Adaboost, adaptive boosting; MLP, multi-layer perceptron; AUC, area under the curve.

For the 10 machine learning models developed, five key metrics were used to assess their generalization performance: accuracy, precision, F1 score, recall, and AUC (area under the curve). These results are summarized in **Table 3**. To facilitate comparison, the five evaluation metrics are visualized as bar charts (**Figure 2**). Additionally, ROC curves for each model are displayed in **Figures 3A, C**.

**Table 3:** Evaluation metrics of all machine learning algorithms.

Model	Models for predicting sepsis					Models for predicting mortality				
	Accuracy	Recall	Precision	F1 score	AUC	Accuracy	Recall	Precision	F1 score	AUC
Decision Tree	0.929	0.937	0.924	0.929	0.952	0.885	0.864	0.902	0.881	0.908
Random Forest	0.945	0.964	0.930	0.945	0.977	0.937	0.930	0.944	0.937	0.972
XGBoost	0.932	0.952	0.918	0.934	0.972	0.942	0.945	0.941	0.943	0.973
KNN	0.926	0.951	0.906	0.928	0.969	0.912	0.945	0.888	0.915	0.963
Naive Bayes	0.864	0.773	0.949	0.851	0.949	0.746	0.665	0.798	0.723	0.810
SVM	0.944	0.945	0.945	0.944	0.975	0.877	0.845	0.904	0.872	0.944
Logistic Regression	0.932	0.939	0.929	0.932	0.971	0.779	0.699	0.836	0.758	0.853
GBT	0.935	0.982	0.899	0.938	0.969	0.952	0.965	0.942	0.953	0.981
Adaboost	0.914	0.928	0.905	0.915	0.960	0.900	0.906	0.896	0.900	0.952
MLP	0.938	0.933	0.945	0.938	0.973	0.869	0.880	0.862	0.870	0.938

XGBoost, extreme gradient boosting; KNN, k-nearest neighbors; SVM, support vector machine; GBT, gradient boosting tree; Adaboost, adaptive boosting; MLP, multi-layer perceptron; AUC, area under the curve.

When sepsis occurrence was used as the outcome variable, the RF model emerged as the best-performing model for predicting sepsis in patients with extensive burns, as it achieved the highest AUC of 0.977. Other performance metrics for this model were similarly strong, with an accuracy of 0.945, a recall of 0.964, a precision of 0.930, and an F1 score of 0.945. As shown in **Figure 3B**, the ROC curve of the RF model significantly outperformed that of a logistic regression model based solely on the SOFA score (z score = 2.689, *P* = 0.007, DeLong’s test (DeLong et al., 1988).

When mortality was used as the outcome variable, the GBT model yielded the highest AUC (0.981), with accuracy (0.952), recall (0.965), precision (0.942), and F1 score (0.953), making it the choice for predicting mortality in patients with extensive burns.

### 3.4 SHAP for the established models

To understand the role of key biomarkers and other variables within the machine learning prediction models, the SHAP tree framework was applied to the best-performing models. SHAP values not only quantify the direct influence of individual biomarkers but also account for their interactions with other variables, providing an interpretable and comprehensive view of model predictions.

**Figure 4:**
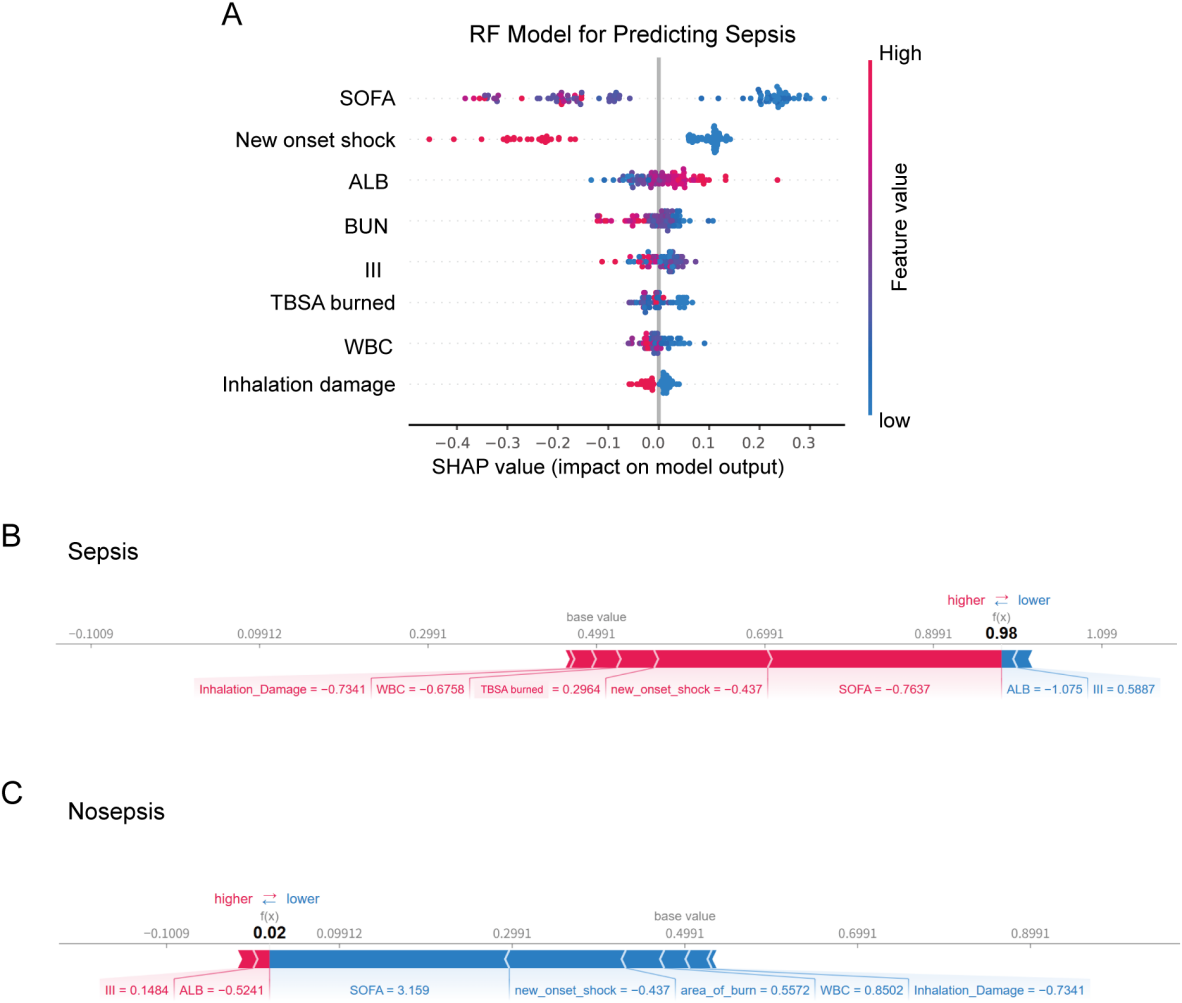
**(A)** SHAP beeswarm summary plot on the impact of input variables on the RF model’s predictive ability. SHAP force plot showing **(B)** high chance of sepsis, **(C)** low risk of sepsis. RF, random forest; SOFA, sequential organ failure assessment; ALB, albumin; BUN, blood urea nitrogen; III, third-degree burned area; WBC, white blood cell count; SHAP, SHapley additive exPlanations.

In the model for predicting sepsis in burn patients, positive SHAP values indicated an increased risk, whereas negative values suggested a protective effect. The greater the SHAP value deviates from 0, the stronger its impact on the prediction. As shown in **Figure 4A**, high albumin levels (indicated by the blue dots) were associated with a lower sepsis risk, whereas high values of features such as SOFA score, new onset shock, ALB, third-degree burned area, WBC, and inhalation injury indicated a higher risk. **Figures 4B, C** present individual SHAP plots for patients with (A) sepsis and (B) non-sepsis. SHAP values highlight the specific predictive features for each individual patient and illustrate the contribution of each feature to sepsis prediction. In **Figure 4B**, red bars indicate features that increase the risk of sepsis, driving the final prediction value f(x) = 0.98, indicating a high risk of sepsis. Notably, the SOFA score, new onset shock and TBSA burned were the most significant factors contributing to increased sepsis risk. In contrast, blue bars represent third-degree burned area, and ALB showed a negative contribution to the risk prediction; however, their effects were relatively small in magnitude compared to the dominant high-risk factors in this specific case. **Figure 4C** shows that while third-degree burned area and ALB slightly increased sepsis risk, the overall effect of most features, particularly those in the blue region, decreased the predicted sepsis risk. Thus, the final prediction value of 0.02 indicated a low risk of sepsis, suggesting that the patient’s overall condition was relatively stable.

**Figure 5:**
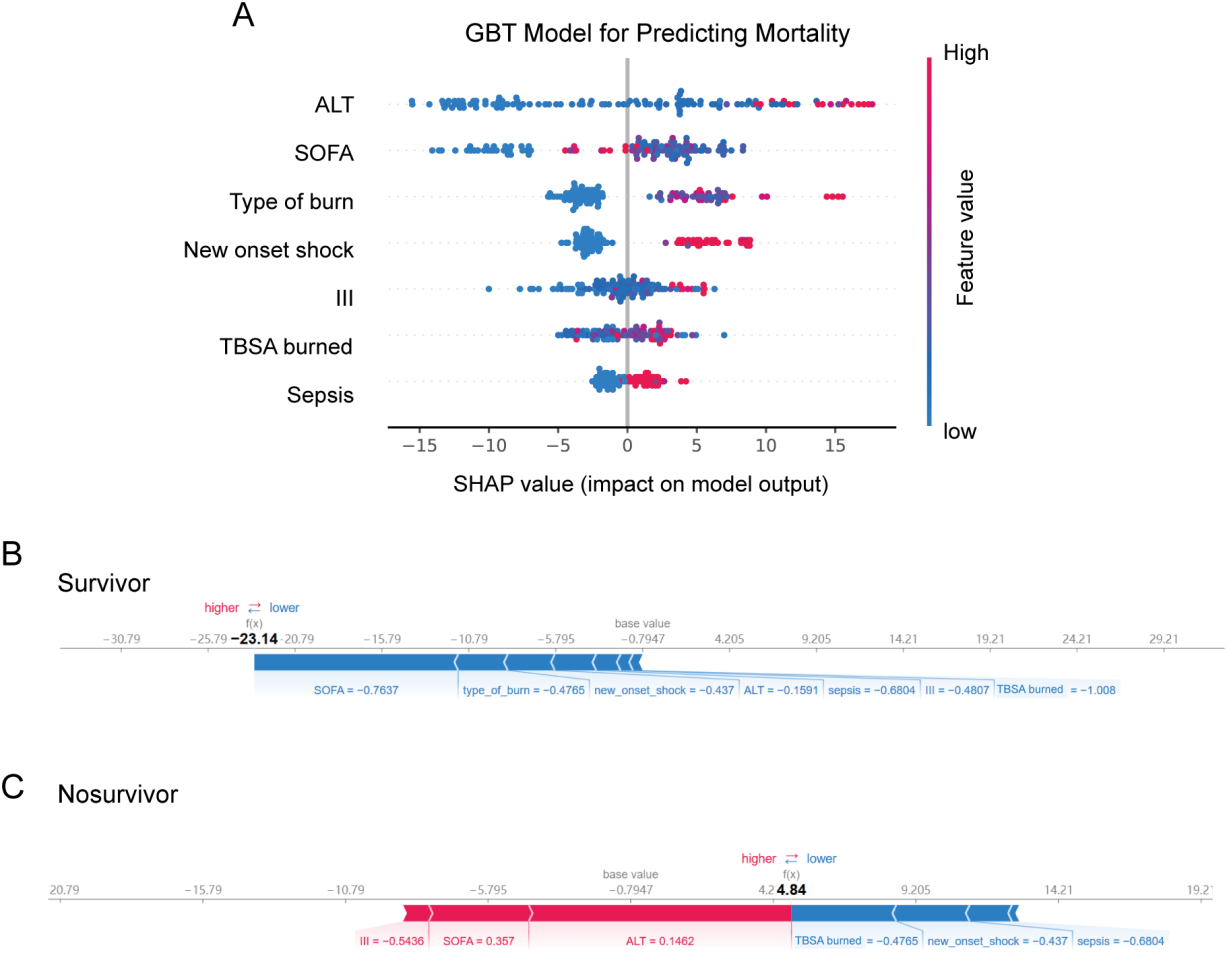
**(A)** SHAP beeswarm summary plot on the impact of input variables on the GBT model’s predictive ability. SHAP force plot showing **(B)** high chance of survival **(C)** low risk of survival. GBT, gradient boosting tree; ALT, alanine aminotransferase; SOFA, sequential organ failure assessment;III, third-degree burned area; SHAP, SHapley additive exPlanations.

**Figure 5A** shows the contributions of biomarkers and clinical features to mortality risk in the GBT model. Elevated levels of ALT, alongside SOFA score and type of burn were the most significant factors driving mortality risk. For surviving patients (**Figure 5B**), certain features, including SOFA score, type of burn, the absence of new onset shock. In deceased patients (**Figure 5C**), a combination of high-risk biomarkers such as third-degree burned area, SOFA score, and ALT drove a high mortality prediction.

### 3.5 Web-based calculators

To facilitate use by healthcare professionals, we developed two web-based calculators via the Streamlit platform. Users can input relevant variables to obtain the predicted probabilities of sepsis and mortality. The calculators are accessible at the two URLs: https://sepsis-prediction-model-for-severe-burn-patients.streamlit.app. And https://mortality-prediction-model-for-severe-burn-patients.streamlit.app.

Examples of how to use the calculators are illustrated in **Supplementary Figure S2** (see the appendix in the electronic **Supplementary Material**).

## 4 Discussion

Managing patients with extensive burns remains clinically challenging due to their high mortality risk and the scarcity of robust clinical data and standardized treatment protocols. In patients with extensive burns, the loss of skin barrier protection, hypovolemia, dysregulated inflammatory responses, and hypermetabolism contribute to a high risk of sepsis, which remains a major cause of mortality in this population. Early identification of sepsis risk factors and the timely application of comprehensive treatments, including antimicrobial therapy, are crucial to improving patient survival. In this study, we collected data from over 12 years of extensive burn patients at two burn centers in Eastern China and used ten machine learning algorithms to develop predictive models for sepsis and mortality based on admission data. Our results indicate that random forest and GBT models performed best for predicting sepsis and mortality, respectively. Through feature importance analysis and SHAP interpretability methods, we identified key risk factors for sepsis and mortality in patients with extensive burns. Finally, the developed web-based tool will assist healthcare workers in applying these findings in clinical practice.

The SOFA score evaluates six critical organ systems, circulatory, respiratory, renal, hepatic, coagulation, and neurological, by incorporating a wide range of parameters, including vasopressor dosage, mean arterial pressure (MAP), respiratory support, serum creatinine, urine output, bilirubin levels, platelet count, and the Glasgow Coma Scale (GCS) score (Singer et al., 2016). As such, it serves as a comprehensive and robust tool for detecting organ dysfunction. In patients with extensive burns, additional factors such as TBSA burned, especially the third-degree burned area, new onset shock, WBC, ALB, BUN, and inhalation injury also play crucial roles. Our comparative analysis indicates that integrating these variables into the model significantly improves its predictive performance for sepsis (**Figure 3B**).

In the models we developed, the TBSA burned emerged as a highly significant biomarker in predicting both sepsis and mortality risk. Studies have shown that the mortality rate for burn patients with a TBSA burned ranging from 21% to 30% is two-thirds higher than that for those with a TBSA burned ranging from 11% to 20% (Lionelli et al., 2005). The survival likelihood decreases markedly with increasing TBSA burned: reported mortality rates reach 51.1%, 70.6%, and 82.6% for patients with TBSA of 70–79%, 80–89%, and ≥ 90%, respectively (Iqbal et al., 2013). In our cohort, where the TBSA burned was ≥ 50%, the mortality rate was 16.03%. Although lower than figures reported in some international studies, the mortality rate falls within the nationally reported range for patients with severe burns (9.79%–23.2%) (Cheng, 2017; He et al., 2022). Several factors may account for this comparatively favorable outcome. First, patients discharged within the first 24 hours were excluded from the analysis; some of these individuals may have been transferred to tertiary burn centers for advanced care or may have discontinued treatment (Cheng, 2017), potentially affecting mortality estimates. Second, a subset of patients sustained extensive burns primarily composed of superficial injuries (e.g., grade I and superficial partial-thickness burns), which inflate TBSA calculations but are associated with better prognoses Finally, data were derived from two specialized burn centers equipped with comprehensive multidisciplinary teams, including intensive care, burn surgery, nephrology, and nutritional support, which likely contributed to improved survival outcomes.

Third-degree burns, also known as full-thickness burns, involve all layers of the skin, leading to severe immune dysregulation (Greenhalgh, 2017). The complete loss of protective barriers significantly increases the risk of both local and systemic infections, thereby increasing the probability of sepsis. Severe fluid loss, electrolyte imbalances, multi-organ failure, and uncontrolled sepsis greatly increase mortality risk. These findings are consistent with previous research reports (Tan Chor Lip et al., 2019; Tsolakidis et al., 2022; Schmidt et al., 2023).

In our study cohort, scald, blast, and electrical burns were associated with higher mortality rates among patients with extensive burns. Although scald injuries typically have lower mortality than flame burns in small-area cases, the risk increases significantly as TBSA increases. For example, among patients in our cohort with scald burns and TBSA ≥ 50%, the mortality rate reached 31.03% (9 deaths out of 29 cases), which is comparable to the 34% reported by Chong et al. in patients with scald injuries over 40% TBSA (Chong et al., 2020). Similarly, Saraçoğlu et al. (Saracoglu et al., 2014) observed a 26% mortality rate in electrical burns with TBSA > 25%, which may be attributed to the high contact temperatures (2000–4000°C) and deep tissue destruction characteristic of electrical injuries.

During the course of illness in burn patients, the occurrence of new onset shock indicates an acute and life-threatening condition. Severe capillary leakage and cardiac suppression result in poor organ perfusion, exacerbating the risk of multiple organ dysfunction. Moreover, the hypermetabolic state induced by burns further depletes the patient’s energy reserves and impairs the immune system, making organ function recovery more challenging. These factors, in combination, significantly impact the prognosis of burn patients (Jeschke et al., 2020).

Among laboratory biomarkers, serum albumin (Alb) has emerged as a strong predictor of sepsis. Lower albumin levels are associated with increased risk (Aguayo-Becerra et al., 2013). Alb exerts a key regulatory role in the inflammatory response by binding to pathogen-associated molecular patterns (PAMPs), such as peptidoglycan, lipoteichoic acid, and lipopolysaccharide, forming Alb–PAMP complexes that modulate immune activation (Gioannini et al., 2002; Esparza et al., 2012). In the pathophysiological context of severe burns, increased capillary permeability and an intensified inflammatory state led to a significant decline in serum albumin levels (Fleck et al., 1985). Hypoalbuminemia, in turn, contributes to extravascular fluid accumulation, resulting in complications such as impaired wound healing and edema (Lehnhardt et al., 2005). Furthermore, decreased albumin levels reduce the body’s antioxidant defenses, rendering patients more susceptible to oxidative stress–related damage associated with sepsis (Taverna et al., 2013). Therefore, in the management of patients with extensive burns, timely supplementation with albumin and blood products may serve as an important strategy for preventing or mitigating sepsis.

WBC count, a classic and sensitive hematological marker for infection, emerged in our model as a stronger predictor of sepsis than PCT. Unfortunately, due to equipment limitations at the participating hospitals, we were only able to collect high-sensitivity CRP (hs-CRP) data for approximately half of the patients. To minimize potential bias, we therefore excluded CRP from the set of predictive variables. Nonetheless, consistency analysis of the available data indicated a high level of agreement between CRP and PCT levels. Both markers are known to be elevated in burn patients (Assicot et al., 1993; Thomas et al., 2004); however, they lack specificity for early sepsis diagnosis in this population (Lavrentieva et al., 2007; Jeschke et al., 2013). By contrast, WBC demonstrated superior predictive ability in our feature selection process. This may be attributed to its greater sensitivity to early systemic inflammatory responses, as well as its broader availability, more frequent testing, and higher temporal stability in clinical settings (Knaus et al., 1985; Rimmer et al., 2022). These findings highlight the potential of WBC as a practical and robust early indicator of sepsis in severely burned patients, especially in resource-limited settings where advanced biomarker testing may not be consistently available.

Inhalation injury has been widely recognized as a significant risk factor for both sepsis and mortality in burn patients (Temiz et al., 2020; Yazıcı et al., 2022). It may result from direct thermal damage to the airway, chemical irritation from toxic gases, and systemic toxicity due to carbon monoxide exposure. These insults can initiate a strong inflammatory response, potentially leading to complications such as pneumonia, acute respiratory distress syndrome (ARDS), and even death. In our cohort of patients with extensive burns, the incidence of inhalation injury was 35.02%, which is slightly higher than the rates reported by most hospitals in China, ranging from 19.09-32.38% (Dou and Zhang, 2021). This relatively high rate may reflect the greater injury severity in our study population. Importantly, 54 out of 83 patients with inhalation injury (65.06%) received timely endotracheal intubation. Although inhalation injury remained a strong predictive factor for sepsis, there was no statistically significant difference in its incidence between survivors and non-survivors. This finding may be partly attributed to the high rate of early airway intervention, which likely mitigated progression to life-threatening respiratory complications. According to expert consensus in China, early prophylactic tracheotomy is recommended for patients at high risk of airway obstruction. Such procedures should ideally be performed before the peak of tissue edema, in order to avoid severe neck swelling that may obscure anatomical structures and increase procedural difficulty (Ming et al., 2018).

BUN, as a marker of renal function (Jeschke et al., 2020), and ALT, as a marker of hepatic injury (Nielson et al., 2017), were both incorporated into the sepsis and mortality prediction models, respectively. Although the SOFA score incorporates key organ function parameters such as creatinine and bilirubin, it remains a categorical scoring system that may overlook subtle variations in laboratory values. The inclusion of continuous variables such as BUN and ALT in the predictive models may therefore capture early or subclinical organ dysfunction that is not adequately represented by SOFA scores alone. This highlights the limitations of SOFA when used as a standalone measure and supports the utility of integrating granular laboratory data for improved risk stratification.

Our study offers several distinctive features that contribute to advancements in predicting sepsis and mortality in critically ill burn patients:

1. Targeted population: We focused on patients with exceptionally extensive burns (TBSA burned ≥ 50%), addressing a critical research gap concerning outcomes in this high-risk population.
2. Comparative machine learning models: We employed and evaluated ten widely used traditional machine learning models to identify the most effective predictive model.
3. SOFA model comparison: We compared models with a single-variable SOFA prediction model, confirming the utility of the SOFA score in predicting sepsis in patients with extensive burns.
4. Model interpretability via SHAP: We utilized SHAP values to interpret the model both globally and on an individual level, enhancing the model’s transparency and clinical applicability.
5. User-Friendly web tools: We developed two accessible web calculators to facilitate usage by healthcare professionals.

However, there are limitations to our study. Although our dataset, which was derived from two burn centers in East China, is representative, its relatively small size may restrict the performance of the machine learning models. Moreover, the limited geographic and demographic scope could affect the generalizability of our findings across diverse regions and ethnic groups. Additionally, we acknowledge that treatment protocols may have evolved over the 12-year period covered in this study (Chai, 2013; Guo et al., 2018; Hunag, 2020). Nevertheless, by applying consistent diagnostic criteria and focusing exclusively on admission-time variables, we aimed to minimize potential confounding effects caused by temporal changes in clinical practice. Finally, the rapid advances in artificial intelligence offer opportunities for further enhancements. Multisensory data, as well as imaging data from CT and MRI, are increasingly being integrated into clinical decision-making (Burns et al., 2020; Gichoya et al., 2022). In future work, we aim to incorporate such data for more precise patient monitoring and timely interventions.

## 5 Conclusion

In patients with extensive burns, the random forest and GBT methods effectively predict sepsis and mortality. By integrating key biomarkers such as TBSA, albumin, total bilirubin, and serum creatinine, these models provide valuable insights into the underlying pathophysiology. The SHAP tool aids in both global and personalized model interpretation, enhancing clinical understanding. Web calculators further simplify usage for medical staff, but their clinical utility requires further validation over time and across independent studies.

## Data Availability

The data analyzed in this study is subject to the following licenses/restrictions: The datasets used and analyzed during the current study are available from the corresponding author on reasonable request. All the code used in this study has been uploaded to https://github.com/ZzZqr/sepsis_and_mortality. Requests to access these datasets should be directed to S-MY, yuansm@163.com.

## Supplementary material

Supplementary Figure 1 Flowchart illustrating the data selection process.

Supplementary Figure 2 Examples of web-based calculators for prediction models in patients with extensive burns. **(A)** The random forest model predicts sepsis.**(B)** The GBT model predicts mortality.
