# Supplementary figures and images for: Development and validation of web-based, interpretable predictive models for sepsis and mortality in extensive burns
Source: Front Cell Infect Microbiol. 2025 Aug 18;15:1586087. doi: 10.3389/fcimb.2025.1586087 (PMC12399588; doi:10.3389/fcimb.2025.1586087)

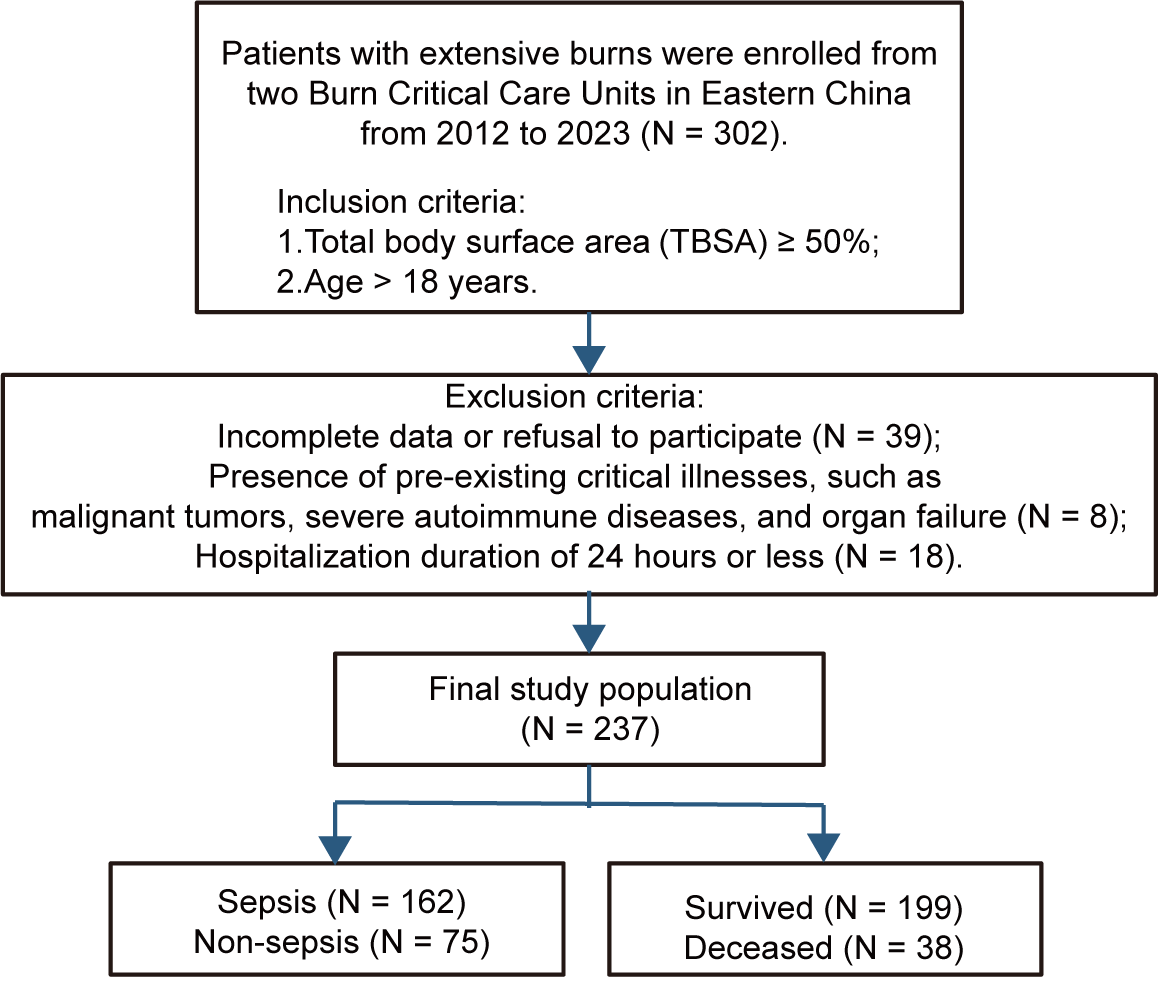

Supplement: Supplementary Figure 1 — Flowchart illustrating the data selection process. [file Image1.tif]

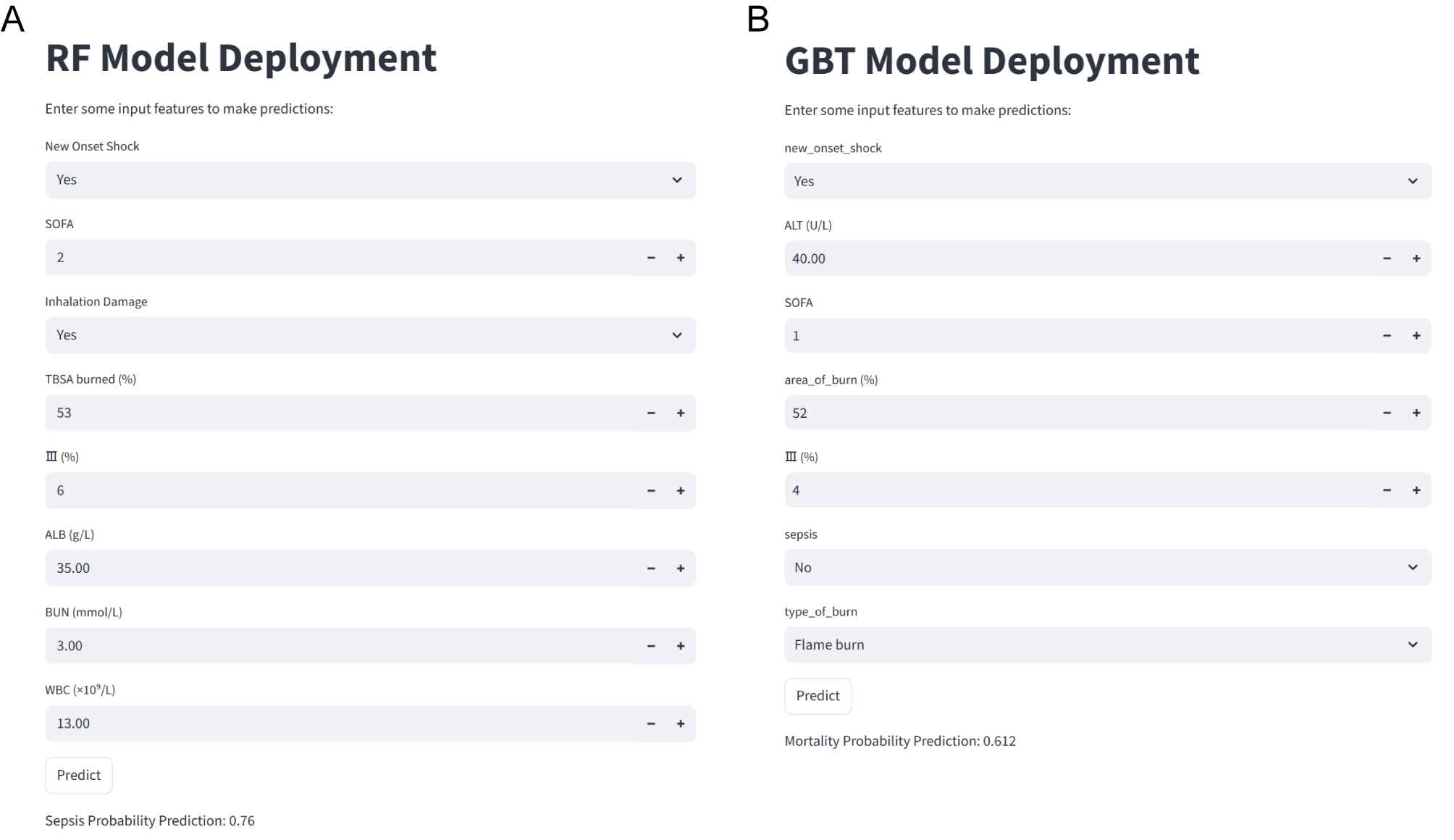

Supplement: Supplementary Figure 2 — Examples of web-based calculators for prediction models in patients with extensive burns. (A) The random forest model predicts sepsis.(B) The GBT model predicts mortality. [file Image2.tif]
