# Supplementary material for: Development and validation of web-based, interpretable predictive models for sepsis and mortality in extensive burns
Source: Front Cell Infect Microbiol. 2025 Aug 18;15:1586087. doi: 10.3389/fcimb.2025.1586087 (PMC12399588; doi:10.3389/fcimb.2025.1586087)
Supplement: Supplementary file 3 [file Table1.docx]

Table S1. In-hospital clinical characteristics: Comparison between sepsis and non-sepsis groups, as well as between survived and deceased groups.

| Variables | n (%) | | | | | |
| --- | --- | --- | --- | --- | --- | --- |
|  | Non-sepsis | Sepsis | *p-*values | Survived | Deceased | *p-*values |
| Wound culture |  |  | <0.001 |  |  | 0.448 |
| No | 89 (54.94) | 21 (28.00) |  | 95 (47.74) | 15 (39.47) |  |
| Yes | 73 (45.06) | 54 (72.00) |  | 104 (52.26) | 23 (60.53) |  |
| Blood culture |  |  | 0.179 |  |  | 0.006 |
| No | 149 (91.98) | 64 (85.33) |  | 184 (92.46) | 29 (76.32) |  |
| Yes | 13 (8.02) | 11 (14.67) |  | 15 (7.54) | 9 (23.68) |  |
| Catheter culture |  |  | 0.470 |  |  | 0.777 |
| No | 147 (90.74) | 65 (86.67) |  | 179 (89.95) | 33 (86.84) |  |
| Yes | 15 (9.26) | 10 (13.33) |  | 20 (10.05) | 5 (13.16) |  |
| MDR |  |  | <0.001 |  |  | 0.05 |
| No | 107 (66.05) | 23 (30.67) |  | 115 (57.79) | 15 (39.47) |  |
| Yes | 55 (33.95) | 52 (69.33) |  | 84 (42.21) | 23 (60.53) |  |
| Surgery times (median, [IQR]) | 0.00 [0.00, 1.00] | 1.00 [0.00, 3.00] | 0.018 | 1.00 [0.00, 2.00] | 0.00 [0.00, 1.75] | 0.141 |
| Tracheotomy time (days) | 1.52 ± 3.92 | 3.61 ± 6.7 | 0.014 | 2.27 ± 5.18 | 1.74 ± 4.33 | 0.503 |
| LOS of hospital (days) | 42.15 ± 2 7.60 | 51.68 ± 31.04 | 0.016 | 49.21 ± 29.00 | 24.00 ± 17.83 | <0.001 |
| MV hour | 3.47 ± 24.19 | 14.65 ± 42.34 | 0.035 | 6.77 ± 30.69 | 8.24 ± 35.54 | 0.813 |

*Abbreviations:*MV, mechanical ventilation; LOS of hospital, length of stay in hospital.
