# Supplementary material for: Development and validation of web-based, interpretable predictive models for sepsis and mortality in extensive burns
Source: Front Cell Infect Microbiol. 2025 Aug 18;15:1586087. doi: 10.3389/fcimb.2025.1586087 (PMC12399588; doi:10.3389/fcimb.2025.1586087)
Supplement: Supplementary file 4 [file Table2.docx]

Table S2. Data distribution before and after SMOTE.

|  | Non-sepsis | Sepsis | Survived | Deceased |
| --- | --- | --- | --- | --- |
| Before over-sampling | 162 | 75 | 199 | 38 |
| After over-sampling | 162 | 162 | 199 | 199 |

*Abbreviations:* SMOTE, synthetic minority oversampling technique.
